# Sex‐Specific Subcutaneous Adipose Tissue Transcriptome in Obesity: Insights From Monozygotic Twin Pairs Discordant for BMI

**DOI:** 10.1002/oby.70078

**Published:** 2025-11-23

**Authors:** Hanna Haltia, Maheswary Muniandy, Sini Heinonen, Sina Saari, Marcus Alvarez, Antti Hakkarainen, Jesper Lundbom, Juho Kuula, Per‐Henrik Groop, Jaakko Kaprio, Päivi Pajukanta, Kirsi H. Pietiläinen, Birgitta W. van der Kolk

**Affiliations:** ^1^ Obesity Research Unit, Research Program for Clinical and Molecular Metabolism Faculty of Medicine, University of Helsinki Helsinki Finland; ^2^ Department of Internal Medicine and Rehabilitation Helsinki University Hospital Helsinki Finland; ^3^ Department of Human Genetics David Geffen School of Medicine at UCLA Los Angeles California USA; ^4^ HUS Medical Imaging Center, Radiology University of Helsinki and Helsinki University Hospital Helsinki Finland; ^5^ Institute for Clinical Diabetology, German Diabetes Center, Leibniz Center for Diabetes Research Heinrich Heine University Düsseldorf Germany; ^6^ Faculty of Medicine, Doctoral Program in Clinical Research University of Helsinki Helsinki Finland; ^7^ Folkhälsan Institute of Genetics Folkhälsan Research Center Helsinki Finland; ^8^ Research Program for Clinical and Molecular Metabolism Faculty of Medicine, University of Helsinki Helsinki Finland; ^9^ Nephrology, Abdominal Center University of Helsinki and Helsinki University Hospital Helsinki Finland; ^10^ Department of Diabetes, Central Clinical School Monash University Melbourne Victoria Australia; ^11^ Institute for Molecular Medicine Finland, FIMM University of Helsinki Helsinki Finland; ^12^ Institute for Precision Health David Geffen School of Medicine at UCLA Los Angeles California USA; ^13^ Healthy Weight Hub, Abdominal Center Helsinki University Hospital and University of Helsinki Helsinki Finland

**Keywords:** adipose tissue, monozygotic twins, obesity, sex differences, transcriptome

## Abstract

**Objective:**

We investigated the impact of sex on the subcutaneous adipose tissue (AT) transcriptome and its obesity‐related adaptations.

**Methods:**

We studied rare BMI‐discordant monozygotic twin pairs (ΔBMI > 2.5 kg/m^2^; 21 female, 16 male pairs) to assess how sex affects AT and whole‐body metabolism. AT RNA sequencing was analyzed using linear mixed modeling and pathway enrichment for: (1) sex differences in individual twins, adjusted for BMI, (2) sex‐stratified effects of acquired obesity (ΔBMI between co‐twins separately in females and males), (3) sex‐specific effects of obesity (differences in the ΔBMI effect between sexes).

**Results:**

(1) AT transcriptional profiles differed between sexes, associating with insulin sensitivity. (2) Sex‐stratified obesity effects within pairs were stronger in females, with upregulated inflammation and downregulated mitochondrial oxidative phosphorylation; males showed increased inflammation and decreased histone modification. (3) The response to obesity was sex‐specific: lower expression of genes in unsaturated fatty acid metabolism in obesity was seen in females only. Sex‐specific obesity AT gene expression was associated with metabolic health, with a negative association between unsaturated fatty acid metabolism and insulin sensitivity in males only.

**Conclusions:**

Biological sex influences the AT transcriptome and its response to obesity, highlighting distinct molecular mechanisms that may contribute to sex‐specific metabolic health.

**Study Importance**

- What is already known?
  ○ Biological sex influences adipose tissue function and metabolism.
  ○ Obesity alters the adipose tissue transcriptome, but sex differences in the effects of obesity remain unclear.
- What does this study add?
  ○ Obesity had a more pronounced altered profile in the female subcutaneous adipose tissue transcriptome compared to males.
  ○ Females with obesity showed lower expression of genes involved in unsaturated fatty acid metabolism, a pattern not seen in males. Notably, these genes were negatively associated with insulin sensitivity in males.
- How might these results change the direction of research or focus of clinical practice?
  ○ Our findings highlight the necessity of incorporating sex‐specific mechanistic studies into human obesity research. Distinct molecular adaptations in AT may help explain sex differences in metabolic disease risk and progression in obesity.
  ○ Future research should explore whether differences in fatty acid gene expression shape lipid profiles, storage, mobilization, and signaling, and how these contribute to sex‐specific metabolic risks in obesity.

## 1 Introduction

Obesity, becoming more prevalent globally, increases the risk for many conditions including type 2 diabetes, multiple cancers, and cardiovascular diseases [1]. Sex plays a crucial role in the development and manifestation of these conditions [2], likely also through its influence on adipose tissue (AT), a key driver of metabolic dysregulation in obesity [3].

AT exhibits distinct sex differences in distribution, cellularity, endocrine control, and lipid metabolism [4]. Females have more AT in subcutaneous depots, whereas males accumulate more visceral AT [4], a pattern associated with differential metabolic risk [2]. Genome‐wide association studies have also revealed sex differences in the relationship between genetic variants and adiposity [5]. Adipocyte size and number also differ, depending on AT depot and study population. Overall, females tend to have more but smaller adipocytes in abdominal subcutaneous AT than males, and adipocyte number increases with BMI in females, but not in males [6]. AT expansion appears more hyperplastic in females and more hypertrophic in males [6]. Females also have better AT insulin sensitivity [7] than males, as well as higher circulating levels of leptin and adiponectin [2]. Moreover, females seem to have lower adipocyte lipolysis [7] and more effectively suppress lipolysis and store fat in AT after a meal [8], but findings depend on metabolic state [8]. These distinct AT characteristics are associated with sex‐specific risks for metabolic complications, including systemic insulin resistance and worse plasma lipid profiles [2].

Subcutaneous AT transcriptome reflects these physiological differences [9, 10, 11], being one of the most transcriptionally divergent tissues between females and males [12]. A large‐scale study identified 162 sex‐differentiated genes, with females showing higher oxidative phosphorylation (OXPHOS) and adipogenesis transcript activity [9]. Similar findings of increased mitochondrial, OXPHOS, tricarboxylic acid cycle, and adipogenesis‐related gene expression in females have been reported in other studies [10, 11]. These sex differences in energy metabolism and AT function have been strongly associated with adiposity, insulin resistance, and plasma lipids [10] and have been suggested to support the preservation of reproductive capacity in females [13].

**FIGURE 1:**
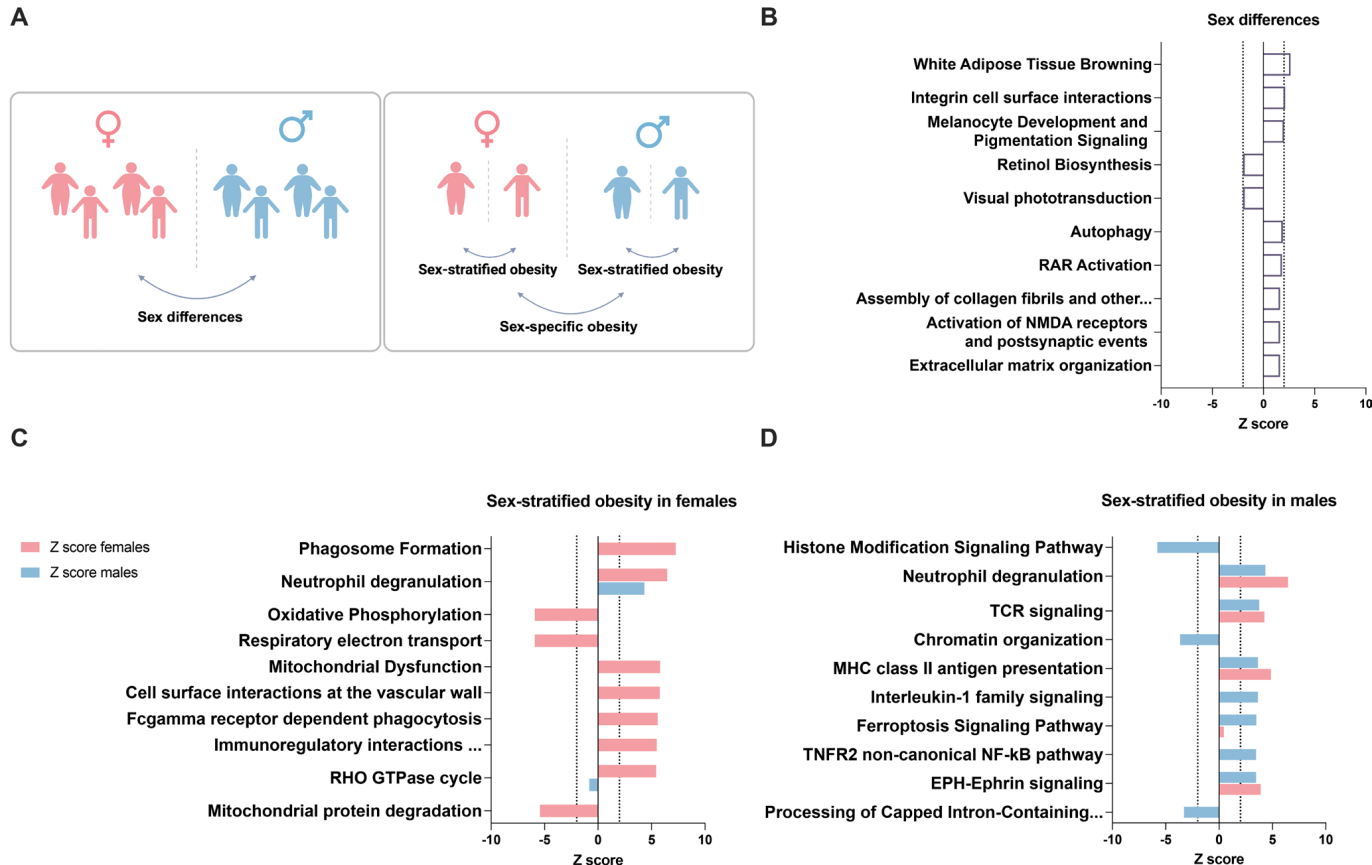
Study design and biological pathway enrichment for sex differences and sex‐stratified obesity differences in adipose tissue gene expression. (A) Overview of the study design using monozygotic BMI‐discordant twin pairs. Two approaches were used: the first assessed sex differences in gene expression by comparing female individuals (*n* = 42) to male individuals (*n* = 32). The second approach examined sex‐stratified obesity by comparing leaner to heavier co‐twins using BMI in females (*n* = 21 pairs) and males (*n* = 16 pairs). Sex‐specific obesity differences in gene expression were obtained by comparing the DEGs from the sex‐stratified comparisons. (B) The top 10 significant (Fisher exact test, highest *z* score) enriched pathways identified by Ingenuity pathway analysis pathways using significant DEGs in males compared to female individuals. (C) The top 10 (highest *z* score) significant (*p* value < 0.05) enriched IPA pathways using DEGs associated with BMI in females. The corresponding *z* score for males is also presented If the pathway was significant in the male analysis. (D) The top 10 significant (highest *z* score) enriched IPA pathways using DEGs associated with BMI in males. The corresponding *z* score for females is also presented If the pathway was significant in the female analysis. IPA provided *z* scores for the pathway directionality by calculating the observed number of “activated” genes (*z* score > 0), “inhibited” genes (*z* score < 0), or pathways with no directionality (*z* score = 0). [Color figure can be viewed at wileyonlinelibrary.com]

In obesity, AT becomes dysfunctional [14], with downregulation of mitochondrial pathways (OXPHOS, branched‐chain amino acid catabolism, fatty acid β‐oxidation) [3, 15], and upregulation of inflammatory [3, 15, 16, 17] and extracellular matrix organization pathways [16, 17]. However, few studies have examined how the obesity‐induced AT transcriptome varies between the sexes, with studies showing lower insulin receptor substrate 1 (*IRS1*) expression in males [7] and differences in chromatin remodeling and lncRNA expression between males and females with obesity [18]. However, previous research has been constrained by small sample sizes, unequal representation of sexes, or by focusing on specific biological pathways or individual genes. Additionally, comparisons of groups with or without obesity are confounded by genetic differences, making it difficult to isolate the specific effects of obesity. To address these, we analyzed how sex and obesity affect the AT transcriptome using a unique BMI‐discordant monozygotic (MZ) twin design, a model setting designed for studying acquired obesity. In three complementary approaches to the AT transcriptome (Figure 1A), we investigated (1) sex differences in individual twins, adjusted for BMI, (2) sex‐stratified effects of acquired obesity (ΔBMI between co‐twins separately in females and males), and (3) sex‐specific effects of obesity (differences in the ΔBMI effect between sexes).

## 2 Methods

### 2.1 Study Participants and Ethics

We recruited 37 BMI‐discordant MZ twin pairs (21 female and 16 male pairs) from population‐based longitudinal studies (FinnTwin16, 2839 pairs [19]; FinnTwin12, 2578 pairs [20]; Older Finnish Twin Cohort, 13,888 pairs [21]). The twin pairs (within‐pair BMI difference, ΔBMI ≥ 2.5 kg/m^2^) had no type 2 diabetes. The study, approved by the Ethics Committee of the Helsinki University Hospital (protocol number 270/13/01/2008), adhered to the Declaration of Helsinki. All participants provided written informed consent.

### 2.2 Study Design

We investigated gene expression alterations in AT in relation to sex and obesity using three complementary approaches (Figure 1A). First, we examined sex differences in the AT transcriptome, comparing female and male twin individuals. Second, we assessed sex‐stratified obesity effects by comparing, separately in each sex, gene expression differences within the BMI‐discordant twin pairs (paired analysis, effects of ΔBMI). Lastly, we identified sex‐specific obesity differences by comparing the effect sizes from the sex‐stratified obesity analyses (compare ΔBMI of females to ΔBMI of males), highlighting within‐pair transcriptional differences unique to each sex in response to obesity.

### 2.3 Anthropometrics and Body Composition

We measured weight, height, waist circumference, whole‐body fat (dual‐energy X‐ray absorptiometry), and abdominal subcutaneous, visceral, and liver fat (magnetic resonance imaging and spectroscopy) [3].

### 2.4 Lifestyle Factors

Alcohol consumption and smoking were assessed by questionnaires and food intake as a 3‐day diary [19, 22]. Physical activity was assessed using the Baecke questionnaire [23].

### 2.5 Clinical Chemistry

Blood was drawn after a 12‐h fast for biochemical analyses (listed in Table 1) using standardized methods at the Helsinki University Hospital laboratories [3].

**TABLE 1:** Clinical variables of the participants for leaner and heavier co‐twins in females and males.

	Females (*N* = 21 pairs)				Males (*N* = 16 pairs)				*p* (ΔF vs. ΔM)
	*n*	Leaner co‐twin	Heavier co‐twin	*p*	*n*	Leaner co‐twin	Heavier co‐twin	*p*	
BMI (kg/m^2^)	21	25.4 ± 5.3	31.4 ± 6.4	**9.54E−07**	16	25.7 ± 3.8	31.3 ± 3.9	**0.003**	0.613
Height (cm)	21	165.6 ± 8.3	165.8 ± 8.4	0.984	16	179.4 ± 7.8	180.4 ± 6.4	0.501	0.902
Weight (kg)	21	69.5 ± 13.7	85.8 ± 17.2	**6.38E−05**	16	83.2 ± 16.7	101.9 ± 15.3	**0.005**	0.163
Waist (cm)	21	82.9 ± 10.6	96.8 ± 12.3	**9.54E−07**	15	91.1 ± 12.7	109.9 ± 10.4	**0.003**	0.198
Age (years)	21	32.7 (24.9–35.4)	32.7 (24.9–35.4)		16	34.1 (32.5–60.1)	34.1 (32.5–60.1)		
Waist‐height ratio	21	0.48 (0.44–0.55)	0.57 (0.52–0.64)	**9.54E−07**	15	0.49 (0.46–0.56)	0.62 (0.56–0.66)	**0.001**	0.534
Waist‐hip ratio	21	0.82 (0.79–0.87)	0.87 (0.83–0.91)	**0.003**	16	0.95 (0.88–1.00)	1.01 (0.99–1.09)	**0.014**	0.172
Body fat (%)	21	35.5 ± 8.6	44.4 ± 6.4	**1.91E−06**	16	28.0 ± 7.5	35.7 ± 4.1	**0.001**	0.421
Subcutaneous fat (cm^3^)	16	3226 (2644–4750)	5946 (4781–8250)	**3.05E−05**	11	3635 (1825–4807)	5070 (4065–7308)	**0.042**	0.422
Intra‐abdominal fat (cm^3^)	16	403 (244–789)	929 (665–1415)	**3.05E−05**	11	962 (677–2246)	2482 (1597–3010)	**0.042**	0.056
Adipocyte volume (pl)	21	383 ± 174	567 ± 209	**9.54E−06**	15	463 ± 137	707 ± 276	**0.017**	0.794
Adipocyte number (10E13)	21	10.2 (9.3–35.2)	8.57 (6.78–60.4)	0.919	15	7.41 (1.74–65.5)	17.6 (7.9–101)	0.013	0.660
Liver fat (%)	16	0.6 (0.4–0.8)	1.0 (0.6–4.1)	**6.10E−05**	11	0.6 (0.4–2.9)	9.1 (1.4–10.4)	0.067	0.089
Aspartate aminotransferase (U/L)	21	25.7 ± 9.3	24.1 ± 5.3	0.809	16	26.4 ± 5.6	29.3 ± 9.1	0.407	0.600
Alanine aminotransferase (U/L)	21	18.0 (13.0–24.0)	22.0 (15.0–27.0)	0.386	16	26.0 (19.3–31.5)	31.0 (19.0–45.5)	**0.034**	0.902
Gamma‐glutamyltransferase (U/L)	16	12.0 (10.0–18.0)	13.5 (10.8–21.0)	0.162	11	21.0 (19.0–27.0)	29.0 (22.0–68.5)	0.328	0.171
Total cholesterol (mmol/L)	21	4.6 ± 0.9	4.7 ± 1.0	0.349	16	4.7 ± 0.9	5.0 ± 0.8	0.334	0.902
LDL cholesterol (mmol/L)	21	2.6 ± 0.7	3.0 ± 0.9	0.058	16	3.0 ± 0.8	3.3 ± 0.7	0.148	0.701
HDL cholesterol (mmol/L)	21	1.8 ± 0.4	1.5 ± 0.4	**0.002**	16	1.5 ± 0.5	1.3 ± 0.5	0.159	0.976
Triglycerides (mmol/L)	21	0.8 (0.8–1.0)	1.1 (0.9–1.3)	**0.026**	16	0.9 (0.7–1.1)	1.2 (1.1–1.8)	**0.034**	0.064
High sensitivity C‐reactive protein (mg/L)	20	1.2 (0.9–2.8)	2.4 (0.9–6.6)	**0.047**	14^a^	0.7 (0.3–1.7)	1.4 (0.6–3.2)	0.153	0.509
Fasting glucose (mmol/L)	21	5.2 ± 0.4	5.3 ± 0.5	0.616	16	5.4 ± 0.5	5.7 ± 0.5	0.155	1.000
Fasting insulin (mU/L)	20	4.7 (3.4–6.4)	7.8 (4.8–10.8)	**0.008**	15	5.0 (3.3–7.0)	7.6 (6.6–9.2)	**0.003**	0.805
HOMA‐IR index	20	1.0 (0.7–1.5)	1.8 (1.0–2.6)	**0.001**	15	1.1 (0.7–1.7)	1.9 (1.7–2.2)	**0.003**	0.705
Matsuda index	20	7.6 (4.9–10.6)	4.8 (3.8–7.4)	**0.005**	15	7.7 (5.5–9.4)	3.9 (3.3–4.9)	**0.002**	0.479
Smoking (number of smokers)	21	5	4	0.766	16	4	4	1.000	0.951
Physical activity (Baecke index)	21	8.4 ± 1.5	8.1 ± 1.3	0.590	15	8.4 ± 1.3	8.3 ± 1.1	0.378	0.100
Alcohol consumption (portions per week)	21	1.0 (0.0–3.0)	2 (0.0–6.0)	0.442	16	3.5 (0.6–9.8)	4.4 (1.8–11.6)	0.834	**0.017**
Total energy intake (kcal)	21	1999 ± 571	2092 ± 593	0.446	16	2169 ± 502	2293 ± 367	0.692	0.847
Protein intake (percentage of total)	21	0.17 ± 0.05	0.16 ± 0.03	0.469	16	0.19 ± 0.03	0.17 ± 0.02	0.218	0.443
Fat intake (percentage of total)	21	0.35 ± 0.08	0.37 ± 0.09	0.303	16	0.36 ± 0.05	0.38 ± 0.05	0.548	0.941
Carbohydrate intake (percentage of total)	21	0.44 ± 0.07	0.43 ± 0.11	0.397	16	0.4 ± 0.07	0.38 ± 0.06	0.557	0.505
Saturated fatty acid intake (percentage of total)	21	0.15 ± 0.04	0.14 ± 0.03	0.458	16	0.14 ± 0.02	0.13 ± 0.03	0.811	0.788
Monounsaturated fatty acid intake (percentage of total)	21	0.11 ± 0.03	0.12 ± 0.04	0.070	16	0.12 ± 0.02	0.12 ± 0.03	0.695	0.183
Polyunsaturated fatty acid intake (percentage of total)	21	0.05 ± 0.01	0.06 ± 0.02	0.008	16	0.05 ± 0.02	0.06 ± 0.02	0.662	0.173

*Note*: Data shown as mean ± SD for normally distributed variables and median (interquartile range) for skewed variables. Heavy‐lean *p* values have been calculated using paired Wilcoxon signed rank exact test, and ΔF versus. ΔM (heavy‐lean comparison in females versus heavy‐lean comparison in males) *p* value has been calculated using Wilcoxon rank sum test. *p* < 0.05 are highlighted in bold.

^a^ Data from two leaner twin participants missing.

### 2.6 AT Biopsy

Superficial subcutaneous abdominal AT biopsies were taken near the umbilicus, under sterile conditions with local anesthesia (lidocaine), using either a surgical or needle biopsy technique. A part of the sample for RNA extraction was snap‐frozen and stored in liquid nitrogen, and part underwent collagenase digestion for adipocyte size measurement [3].

### 2.7 RNA Sequencing

Total RNA was extracted from ~250 mg of frozen AT using the AllPrep Universal Kit (QIAGEN, Sollentuna, Sweden) with a DNAse I (QIAGEN) digestion according to the manufacturer's instructions. RNA quality control was assessed with a 2100 Bioanalyzer (Agilent Technologies, Santa Clara, CA, USA), with all samples showing RIN > 8.

We prepared the libraries using Illumina Stranded mRNA preparation (Illumina Inc., San Diego, CA, USA) and sequenced the samples using the Illumina HiSeq2000 (Illumina) platform to an average sequence depth of 40–50 million paired end reads (75 bp). We aligned the reads to hg38 using STAR v2.5.2b and its two‐pass protocol with Gencode v26 annotations [24]. Samples required ≥ 20 M uniquely mapped reads and correct strandedness. Quality was assessed using Picard [25], and genotype‐RNA sequencing concordance was verified using exonic SNPs with VerifyBamID [26]. Read counts were calculated using HTSeq [27] v0.6.1p. We retained genes with ≥ 10 counts in ≥ 70% of samples.

### 2.8 Statistical Analysis

#### 2.8.1 Participant Characteristics

Normality of anthropometric and clinical variables was determined using histograms and Shapiro–Wilk tests. Data are expressed as mean ± standard deviation (SD) for normally distributed variables and as median (interquartile range) for non‐normal variables. (1) Sex differences: female individuals were compared to male individuals using linear mixed modeling (lme4 [28]) with twinship accounted for as random effects. (2) Sex‐stratified obesity (within‐pair): heavier and leaner co‐twins were compared using paired Wilcoxon signed rank test separately within female and male twin pairs. (3) Sex‐specific obesity effect: female Δheavier–leaner was compared to male Δheavier–leaner with Wilcoxon rank sum test. We considered *p* < 0.05 statistically significant. All data were analyzed using R v4.4.2.

#### 2.8.2 Differential Gene Expression Analysis

1. For the sex differences in AT transcriptome, we normalized the data (edgeR [29]) and did a differential global gene expression analysis to identify differentially expressed genes (DEGs) in male compared to female individuals (Figure 1A) using a linear model (limma [30]). In this multilevel model, correlation between twins is used to represent the genetic similarities between co‐twins. We used BMI, smoking status, age, and batch effect adjustments.
2. For sex‐stratified obesity (Figure 1A), we analyzed associations between ΔBMI and gene expression, separately within female and male twin pairs. We used a paired‐sample analysis model adjusted for smoking status that identifies only the nongenetic and nonshared environmental factors that associate with BMI between the co‐twins. In this paired design, factors like age and genetics were inherently controlled for within twin pairs.
3. For sex‐specific obesity (Figure 1A), using the same model as in sex‐stratified obesity, we compared the effects of obesity (effect of BMI in females versus males). From this contrast, we obtained the DEGs identified in our sex‐stratified obesity analysis that differed between the two sexes. The sex‐specific DEGs were divided into four lists: DEGs upregulated with greater BMI in both female and male twin pairs, DEGs downregulated with greater BMI in both female and male twin pairs, DEGs that, with greater BMI, were upregulated in male twin pairs but downregulated in female twin pairs or vice versa. Multiple testing was controlled with the Benjamini–Hochberg method (FDR < 0.05).

#### 2.8.3 Biological Pathway Analysis

For sex differences and sex‐stratified obesity, we analyzed the DEGs using Ingenuity pathway analysis (Ingenuity Systems, Redwood City, CA, USA, v24.0). For sex‐specific obesity, we performed separate functional enrichment analysis with each DEG list using Reactome pathways [31] via overrepresentation analysis with WebGestalt [32]. The overrepresentation analysis is based on Fisher's exact test, comparing the gene list of interest to the full set of sequenced transcripts. Multiple testing was controlled with the Benjamini–Hochberg method (FDR < 0.05).

#### 2.8.4 Clinical Association Analyses

To assess the clinical relevance of the DEGs related to sex differences and sex‐specific obesity, we took the pathway scores of the most prominent biological pathways/networks, resulting in six scores, and used linear regression to study them in relation to clinical parameters. From the sex differences analysis, we selected the top two Ingenuity pathway analysis pathways with the highest absolute *z* scores: “white adipose tissue browning” and “integrin cell surface interactions.” From sex‐specific obesity, we created four scores based on DEGs that belonged to the same biological network, for example, reactome pathways with similar biological functions, which were “other interleukin signaling,” “transcription,” “fatty acid metabolism,” and “proteasome.” Pathway scores were calculated by standardizing gene expression levels (mean = 0, SD = 1 across all individuals) and averaging the standardized values. Linear mixed models (lme4 [28]) were used to assess the association between pathway scores and clinical parameters in individuals. We adjusted the models for age, smoking, and BMI.

## 3 Results

### 3.1 Sex Differences: Comparing Individual Females and Males

Mean BMI was similar between females and males (Table S1). However, males had a higher waist‐hip ratio, intra‐abdominal and liver fat, liver enzymes alanine aminotransferase (ALAT), gamma‐glutamyltransferase (GT), fasting glucose, and triglycerides. In contrast, females had a higher body fat percentage. Insulin sensitivity (HOMA‐IR and Matsuda indices) and smoking frequency were comparable between the sexes. Adipocyte size and dietary intake of macronutrients were similar between females and males.

We identified 17,537 transcripts, of which 513 were significant DEGs between the sexes in the AT transcriptome, the majority (316) upregulated in male compared to female individuals (Table S2). The most prominent pathways distinguishing the sexes were white adipose tissue browning (*z* score 2.7) and integrin cell surface interactions (*z* score 2.1), which were predicted to be more active in males than in females (Figure 1B, Table S8). Additionally, retinoid‐related metabolism tended to differ by sex, with lower retinol biosynthesis and visual phototransduction (*z* score −2.0 for both) and higher RAR–activation (*z* score 1.8) in males.

### 3.2 Obesity: Sex Stratified Results From Comparing Co‐Twins in BMI Discordant Pairs

In sex‐stratified analyses, we investigated how acquired obesity affects the AT transcriptome within female and male pairs (Figure 1A). As per study design, in both female and male pairs, the heavier co‐twins had more adiposity (% body fat, subcutaneous, intra‐abdominal) and a higher waist‐hip ratio. The heavier co‐twins also had worse metabolic health (insulin resistance, triglycerides, high‐sensitivity C‐reactive protein) and larger adipocytes than their leaner co‐twin (Table 1). Additionally, HDL levels were lower in the heavier co‐twins in the female pairs. There were no differences in dietary intake of macronutrients between co‐twins in either females or males.

#### 3.2.1 Females Showed More DEGs in Obesity and Higher Pathway Activation Scores in Response to Obesity (ΔBMI)

Within female twin pairs, 2334 DEGs were associated with greater ΔBMI (1354 upregulated, 980 downregulated; Table S3), while males had 1655 DEGs (626 upregulated, 1029 downregulated). The predicted pathway activation scores, as indicated by pathway *z* scores, were higher in female (Figure 1C) compared to male twin pairs (Figure 1D). These results suggest that BMI‐related differences between co‐twins, i.e., the effects of obesity independent of genetic background, have a greater impact on the female than the male transcriptome.

#### 3.2.2 Obesity in Females Was Linked to Higher Inflammation and Lower OXPHOS Expression

In female twin pairs, ΔBMI DEGs (i.e., the effect of acquired obesity) were primarily associated with upregulated inflammatory pathways and downregulated OXPHOS and respiratory electron transport pathways (Figure 1C). Among the top 10 pathways, inflammatory pathways were upregulated, including phagosome formation (*z* score 7.3) and neutrophil degranulation (*z* score 6.5) (Figure 1C). Notably, all three downregulated pathways among these top pathways were mitochondria‐related, such as OXPHOS (*z* score −5.9) and mitochondrial protein degradation (*z* score −5.4), suggesting a shift toward mitochondrial impairment in the AT profile in obesity in females.

#### 3.2.3 Obesity in Males Was Linked to Decreased Transcriptional Regulation and Increased Inflammation and Programmed Cell Death

In the male twin pairs, ΔBMI DEGs showed downregulated transcriptional regulation (histone modification: *z* score −5.8, chromatin organization: *z* score −3.7) and upregulated inflammation (neutrophil degranulation: *z* score 4.3) and programmed cell death pathways (ferroptosis signaling: *z* score 3.5), indicating impacts on cellular integrity and immune response (Figure 1D).

Neutrophil degranulation (*z* scores: female 6.5; male 4.3) was the only pathway among the top 10 shared between females and males. This indicates that AT inflammation in obesity occurs in both sexes, further supported by the upregulation of several other inflammatory pathways in both sexes (Tables S4 and S5).

### 3.3 Sex‐Specific Obesity: Results From Comparing Sex‐Stratified Co‐Twin Analysis

**FIGURE 2:**
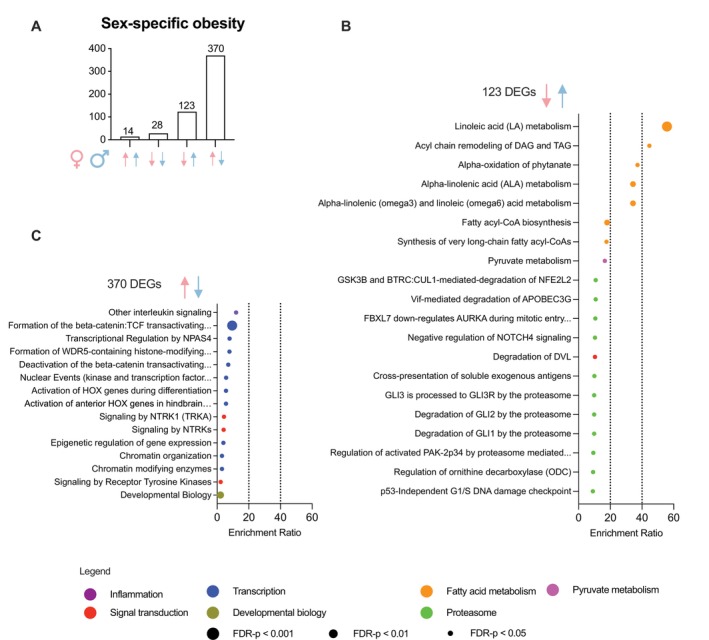
Biological pathways for sex‐specific obesity‐related DEGs in adipose tissue. (A) Significant DEGs from the sex‐specific obesity comparison were categorized into four groups based on their expression pattern in males and females. An upward arrow means higher expression and downward arrow lower expression associated with BMI. (B) Reactome pathways enriched among BMI‐associated genes that were upregulated in males and downregulated in females. (C) Reactome pathways enriched among BMI‐associated genes that were upregulated in females and downregulated in males. [Color figure can be viewed at wileyonlinelibrary.com]

Next, we specifically examined ΔBMI DEGs that showed significant sex differences. Despite similar ΔBMI (females 5.9 vs. males 5.6 kg/m^2^), Δweight (females 16.3 vs. males 18.7 kg) and Δdietary intake of macronutrients between female and male twin pairs (Table 1), we identified 534 sex‐specific obesity DEGs. These DEGs were divided into four distinct groups based on whether they were upregulated or downregulated in obesity (with greater BMI) in female and male twin pairs (Figure 2A). Most DEGs showed opposing patterns—upregulated in males but downregulated in females or vice versa—rather than shared patterns, where genes were consistently up‐ or downregulated in both sexes with greater ΔBMI (Figure 2A). Of the opposing sex‐specific obesity DEGs, 123 transcripts (23%) were upregulated in males but downregulated in females, while 370 transcripts (69%) showed the reverse pattern.

The top overrepresented pathways among sex‐specific obesity DEGs that were upregulated in males but downregulated in females with greater ΔBMI were related to fatty acid metabolism, including pathways such as linoleic acid metabolism (enrichment score [ES]: 56), di‐ and triacylglycerol remodeling (ES: 45), and alpha‐linolenic acid metabolism (ES: 34) (Figure 2B). Additionally, the upregulated male but downregulated female pattern was seen in several pathways involved in the ubiquitin‐proteasome system and transcriptional regulation pathways (Figure 2B).

For the sex‐specific obesity DEGs upregulated in females but downregulated in males with greater ΔBMI, the top enriched pathways included inflammatory signaling (i.e., interleukin signaling, ES: 11.9), several transcriptional regulation pathways (e.g., beta‐catenin transactivating complex formation and deactivation, ES: 9.5 and 7.0, respectively), and several chromatin remodeling pathways (e.g., epigenetic regulation of gene expression, ES: 3.9) (Figure 2C, Table S7). Notably, the enrichment scores in this category (Figure 2C) were substantially lower than in the vice versa comparison (Figure 2B), indicating less pronounced sex‐specific differences.

#### 3.3.1 Unsaturated Fatty Acid Elongation and Desaturation Genes Were Obesity‐ and Sex‐Specific

**FIGURE 3:**
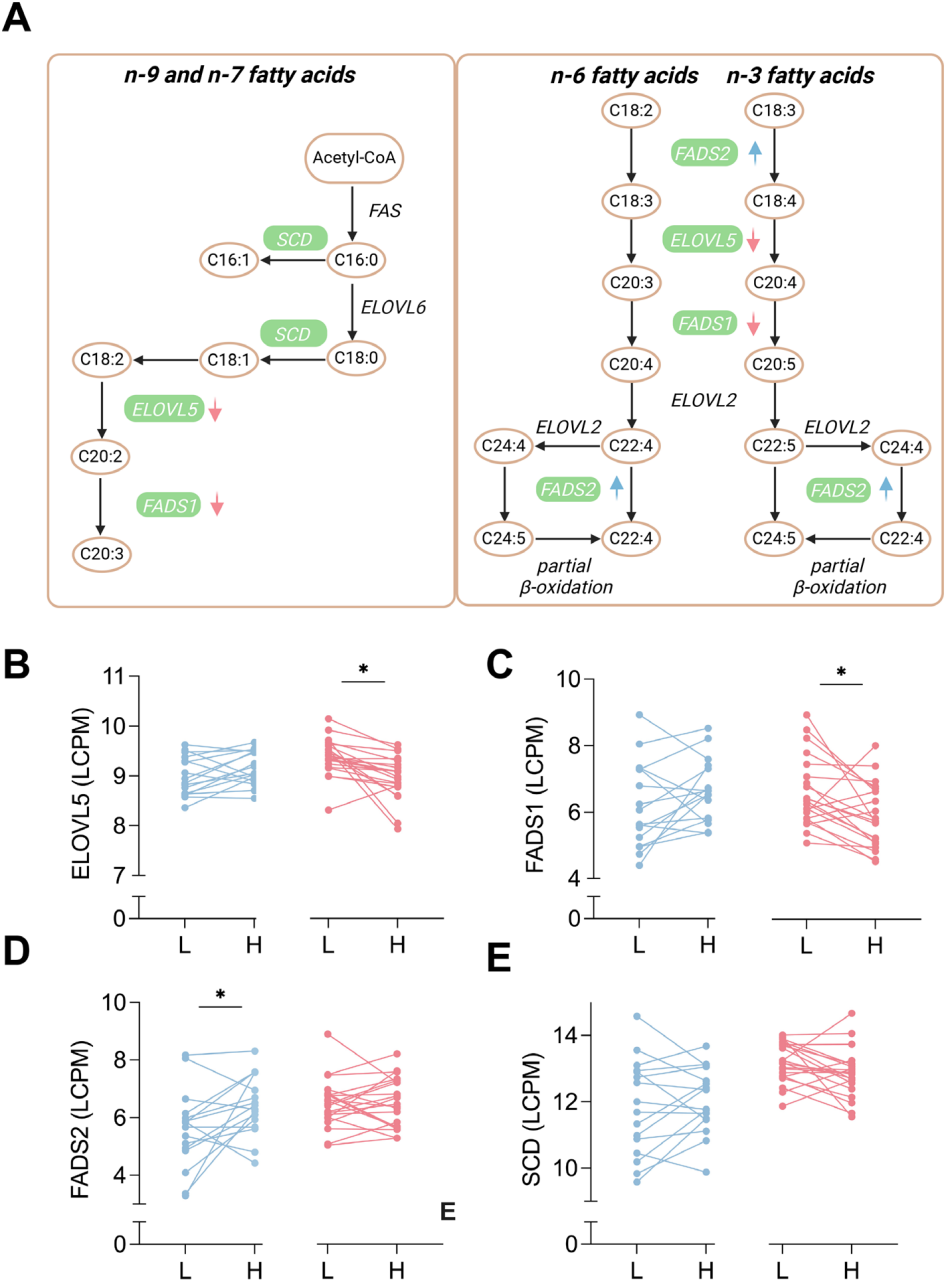
Sex‐specific obesity differences in adipose tissue fatty acid metabolism gene expression. (A) Fatty acid elongation and desaturation pathways, highlighting genes with a sex‐specific expression difference associated with BMI in green. An upward arrow indicates higher expression and downward arrow lower expression associated with BMI. Pink arrows represent expression patterns in females, while blue arrows represent males. (B–E) Log counts per million (LCPM) values showing the expression of four key fatty acid metabolism genes in leaner and heavier co‐twins, separated by sex (blue for males, pink for females). Asterisks indicate significant DEGs associated with BMI: * FDR‐*p* < 0.05. [Color figure can be viewed at wileyonlinelibrary.com]

A closer examination of DEGs with a sex‐specific obesity difference revealed that genes important for fatty acid elongation and desaturation were differentially expressed (Figure 3A). Genes involved in fatty acid elongation (e.g., *ELOVL5*, Figure 3B) and desaturation (e.g., *FADS1*, Figure 3C) were significantly downregulated in obesity in females, but not in males. Conversely, *FADS2*, another desaturation isoform, was upregulated in obesity in males but had a similar expression pattern in female twin pairs (Figure 3D). Notably, *SCD*, a key enzyme in fatty acid desaturation, showed a significant opposing transcription pattern in obesity in females vs. males but was not a DEG in the sex‐stratified co‐twin analysis (Figure 3E).

#### 3.3.2 Fatty Acid Metabolism Gene Expression Associated With Insulin Sensitivity in Males, but Not Females

To examine whether the observed DEGs and their associated pathways relate to clinical phenotypes, we associated six pathway scores, based on the most prominent pathways from the sex differences and sex‐specific obesity analyses, with clinical variables.

Based on the sex differences analysis, we selected the top pathways white AT browning and integrin cell surface interactions. The white AT browning pathway score showed a sex‐specific association for body fat percentage (FDR *p* = 0.018), with males having a tendency for a positive association (FDR *p* = 0.078) and females showing no significant association (Table 2) and fasting insulin (FDR *p* = 0.038) but no significant association within males or females. The integrin cell surface interactions pathway score showed a sex‐specific association for HDL (FDR *p* = 0.004) and ALAT (FDR *p* = 0.035). Of these, the integrin pathway score was associated negatively with HDL in males, but not for ALAT, and none was associated significantly in females (Table 2).

**TABLE 2:** Associations between sex differences in AT gene expression with anthropometric and clinical variables.

Pathway	Clinical variable	*N*	Females			Males			Sex‐specific *p*
			*β*	95% CI	FDR	*β*	95% CI	FDR	
White adipose tissue browning	Body fat %	74	−0.11	−0.485, 0.302	0.978	0.73	0.114, 1.291	0.078	**0.018**
White adipose tissue browning	Fasting insulin	70	0.46	−0.21, 1.14	0.427	−0.85	−1.817, 0.12	0.485	**0.038**
Integrin cell surface interactions	HDL	74	0.22	−0.197, 0.637	0.692	−0.83	−1.402, −0.277	**0.037**	**0.004**
Integrin cell surface interactions	Alanine aminotransferase	74	−0.10	−0.665, 0.446	0.743	0.90	0.184, 1.607	0.130	**0.035**
Fatty acid metabolism	Total cholesterol	74	−0.26	−0.667, 0.132	0.455	0.58	0.098, 1.084	0.173	**0.016**
Fatty acid metabolism	Matsuda index	70	−0.05	−0.535, 0.45	0.918	−0.89	−1.381, −0.381	**0.007**	**0.025**
Fatty acid metabolism	LDL	74	−0.26	−0.676, 0.137	0.448	0.44	−0.059, 0.946	0.357	**0.047**

*Note*: Top pathways and pathway groups from the sex difference and sex‐specific obesity analyses, representing distinct biological functions, were identified. For each pathway (group), we derived a pathway score and performed a linear regression analysis to study the associations of pathway scores with clinical variables. The model was adjusted for age, smoking, and BMI. *p* < 0.05 was considered statistically significant, and significant results are shown in bold. This table shows only pathway scores with significant sex‐specific associations; full results are available in Table S9.

From the sex‐specific obesity pathway scores, only the pathway score for fatty acid metabolism showed sex‐specific associations to clinical variables (Table 2 and Table S8), particularly for total cholesterol, LDL, and Matsuda index (all FDR *p* < 0.05). In males, a higher fatty acid metabolism pathway score was associated negatively with Matsuda index (FDR *p* = 0.007), but not in females. Total cholesterol and LDL did not show significant sex‐stratified associations. These results suggest that sex‐specific gene expression patterns in unsaturated fatty acid metabolism pathways may influence metabolic health in obesity, especially when related to insulin resistance.

## 4 Discussion

This study provides new insights into how sex influences the subcutaneous AT transcriptome, its response to obesity, and how it relates to metabolic health. Using BMI‐discordant MZ twin data, we analyzed AT gene expression in three contexts: (1) sex differences in individual twins, (2) sex‐stratified effects of acquired obesity (ΔBMI between co‐twins separately in females and males), and (3) sex‐specific effects of obesity (differences in the ΔBMI effect between sexes).

Our findings reveal that the AT transcriptome differs significantly between sexes independent of BMI, with males showing higher white AT browning pathway activity and altered retinoid signaling patterns. Moreover, obesity affects the AT transcriptome more in females, with lower mitochondrial OXPHOS expression and increased inflammation, whereas males show reduced transcriptional regulation and increased inflammation and cell death pathways. We also show that genes regulating unsaturated fatty acid metabolism were downregulated in females with obesity, but not in males, suggesting sex‐specific lipid processing in obesity. Importantly, this sex‐specific obesity gene expression pattern in unsaturated fatty acid metabolism was associated with metabolic health, particularly in insulin‐resistant males.

First, we identified 513 DEGs between female and male individuals, a number consistent with previous findings [30, 33, 34] but lower than some earlier reports [12]. These sex difference DEGs were enriched for upregulated white AT browning and downregulated retinol biosynthesis in males. Intriguingly, while the white AT browning pathway is known for mitochondrial metabolism, DEGs within this pathway were not related to mitochondria, but the top upregulated DEGs were cAMP‐ and AMPK‐related genes, key signaling molecules involved in several downstream processes, including adipocyte lipolysis activation [8]. While some studies report higher subcutaneous AT [8] or adipocyte [7] lipolysis in males with obesity [25], sex differences vary by study design, AT depot, and metabolic state (fasting, postprandial, or exercise) [8]. However, as we did not directly assess lipolysis, metabolic states, or retinol levels, the functional impact of these transcriptomic differences remains uncertain.

Second, our sex‐stratified analysis of ΔBMI‐related DEGs revealed that obesity had a stronger impact on the female AT transcriptome. Females exhibited more DEGs (2334) and higher pathway activation scores (up to |*z* score| 7.7) per unit increase in ΔBMI within the twin pairs than males (1655 DEGs and highest pathway |*z* score| 5.8). Obesity in females was characterized by upregulated inflammatory pathways and a downregulated mitochondrial OXPHOS pathway, consistent with findings from all‐female [35] and mixed‐sex studies [3]. Obesity in males was associated with decreased transcriptional regulation (e.g., histone modification) and increased activation of several inflammatory pathways. While the mechanisms behind upregulated inflammation remain unclear, one plausible explanation involves a disrupted lipid metabolism in obesity. In males, one of the upregulated pathways was ferroptosis signaling which reflects a form of ROS‐driven cell death, suggesting that polyunsaturated fatty acid (PUFA) oxidation may contribute to membrane damage and inflammation [36].

Supporting this hypothesis, our sex‐specific obesity analysis (ΔBMI effect in females versus males) revealed a distinct regulation of genes involved in unsaturated fatty acid metabolism. Females typically have a lower proportion of saturated and a higher proportion of unsaturated fatty acids in AT than males [37, 38, 39], though this varies with population [38, 39], hormonal state, and dietary intake [40]. In females, obesity was linked to downregulated fatty acid elongation (*ELOVL5*) and desaturation (*FADS1*) gene expression, theoretically suggesting lower PUFA content and potentially lower lipid peroxidation susceptibility [41]. In contrast, *FADS2* was upregulated in males with obesity. Although sex‐specific obesity effects on these genes have not been directly studied, previous research found higher *FADS1* [9], *SCD*, and *ELOVL5* [42] gene expression in female individuals across both gluteal and abdominal subcutaneous depots. Despite suggesting a sex‐specific obesity regulation of unsaturated fatty acid metabolism, we lack AT fatty acid composition data to connect the transcriptome to the lipidome. We did not find sex differences nor sex‐specific obesity differences in dietary fat intake. In our previous BMI‐discordant MZ twin cohort, obesity was generally linked to higher levels of long‐chain PUFAs in AT [43], but sex‐specific obesity effects were not analyzed. Whether PUFA levels or their peroxidation potential are indeed higher in male AT in the context of obesity remains for future studies. However, a general increase in long‐chain PUFAs in AT in obesity is apparent [43].

Another sex‐specific obesity finding in AT was chromatin remodeling, in line with Rey et al. [18]. DEGs upregulated in female obesity but downregulated in males included lysine methyltransferases (*KMT2B*, *KMT2D*, *KMT2E*) and lysine demethylase (*KMD6B*). Obesity is broadly linked to epigenetic modifications [44], and adipogenesis has also been linked to histone methyltransferase activity [45]. These differences suggest sex‐specific epigenetic regulation of AT remodeling in obesity, though further studies are needed to confirm their functional impact.

Finally, we highlight the importance of the AT transcriptome in metabolic health. Previously, we linked low mitochondrial OXPHOS and high inflammatory activity in AT to obesity‐related complications—such as high liver fat, insulin resistance, and dyslipidemia [3]. Here, we show that the AT transcriptome is associated in a sex‐specific way with insulin sensitivity, lipid profiles, and body fat percentage despite similar BMI and weight differences within pairs. The most striking finding is that the pathway score for fatty acid metabolism is associated with insulin sensitivity in males, but not in females. This sex‐specific association suggests that alterations in fatty acid metabolism may play a more direct role in shaping insulin action in male AT. Indeed, prior studies link greater AT insulin resistance in males with obesity compared to males without obesity to lower *IRS1* expression, higher basal lipolysis, and weaker insulin‐mediated lipolysis suppression [7]. Together, these findings may indicate that altered unsaturated fatty acid metabolism affects lipolytic activity and insulin signaling in a sex‐specific manner, specifically in males. Moreover, our results support the notion that sex‐specific molecular mechanisms in AT may explain the higher metabolic disease susceptibility that is observed in males, both with and without obesity [2].

The major strength of our study is the use of a BMI‐discordant MZ twin design, which provides a genetically controlled framework for exploring sex‐specific gene expression differences in obesity. This design controls for genetic background and early‐life environmental influences, allowing us to attribute differences in transcriptome patterns to acquired obesity rather than hereditary factors. The primary limitation associated with this study is its cross‐sectional nature. Although we can exclude genetic and shared early environmental factors from the observed associations, our cross‐sectional design prohibits causal inferences. We also lack direct functional validation of these gene expression differences such as histology and AT fatty acid composition, and our dietary intake assessment is limited to 3‐day food diaries.

## 5 Conclusion

In conclusion, our findings reveal both BMI‐independent sex differences and distinct obesity‐driven transcriptome profiles in AT between sexes, with more pronounced alterations in females in obesity. Notably, lower expression of genes involved in unsaturated fatty acid metabolism was seen in females in obesity, a pattern not observed in males. Additionally, our findings provided valuable sex‐specific insights into metabolic health, particularly for insulin sensitivity. Overall, our study highlights sex‐specific AT differences in obesity, offering new insights into molecular pathways that distinguish females and males.

## Author Contributions

Maheswary Muniandy, Kirsi H. Pietiläinen, and Birgitta W. van der Kolk conceptualized the study. Sini Heinonen, Jaakko Kaprio, and Kirsi H. Pietiläinen participated in the original twin study design and collected the clinical study data. Päivi Pajukanta generated the RNA sequencing data. Marcus Alvarez and Päivi Pajukanta participated in the RNA sequencing analysis. Hanna Haltia and Maheswary Muniandy performed the statistical analyses as follows: Hanna Haltia and Maheswary Muniandy analyzed the clinical parameters; Maheswary Muniandy performed the transcriptomics analyses; Hanna Haltia performed the pathway analyses; Maheswary Muniandy examined the associations between the AT gene expression with clinical parameters. Antti Hakkarainen, Jesper Lundbom, Juho Kuula, and Per‐Henrik Groop participated in the imaging of the twins. Hanna Haltia, Maheswary Muniandy, Kirsi H. Pietiläinen, Birgitta W. van der Kolk, and Sina Saari contributed to the data interpretation. Maheswary Muniandy, Kirsi H. Pietiläinen, and Birgitta W. van der Kolk supervised the work. Hanna Haltia, Maheswary Muniandy, and Birgitta W. van der Kolk wrote the manuscript. All authors revised the contents of the manuscript and read and approved the manuscript before submission and publication.

## Conflicts of Interest

The authors declare no conflicts of interest.

## Supporting information


**Table S1:** Clinical variables of the participants compared between the female and male individuals.
**Table S2:** Differentially expressed genes between female (*N* = 42) and male (*N* = 32) participants.
**Table S3:** Differentially expressed genes for sex stratified obesity and sex‐specific obesity in BMI‐discordant twin pairs.
**Table S4:** Biological pathways for adipose tissue differentially expressed genes in the sex differences analysis.
**Table S5:** Biological pathways for adipose tissue differentially expressed genes in the sex stratified analysis for females.
**Table S6:** Biological pathways for adipose tissue differentially expressed genes in the sex stratified analysis for males.
**Table S7:** Reactome pathways using Webgestalt overrepresentation analysis using the differentially expressed genes from the sex‐specific obesity comparison.
**Table S8:** Pathways and biological entities used in the clinical association analyses.
**Table S9:** Associations between sex differences in AT gene expression with anthropometric and clinical variables.

## Data Availability

RNA sequencing data from the Twin Study is deposited in the THL Biobank (ID: THLBB2021_001). Bona fide researchers can apply for access via THL Biobank's application process.
